# Associations between family cohesion, stress and digital self-regulation in young adults

**DOI:** 10.3389/fpsyg.2026.1842874

**Published:** 2026-05-08

**Authors:** Büşra Ökten

**Affiliations:** Independent Researcher, Istanbul, Türkiye

**Keywords:** digital self-regulation, digital well-being, family cohesion, life satisfaction, psychological stress, young adults

## Abstract

**Introduction:**

The widespread of digital technologies has transformed family communication, stress coping, and emotional bonds during young adulthood. This study examines relationships between family cohesion, psychological stress, and digital self-regulation, focusing on how family bonds shape digital behavior beyond individual control.

**Methods:**

Using a cross-sectional design, data were collected from 406 university students in Turkey via an online survey and analyzed using structural equation modeling (SEM).

**Results:**

Findings indicate that family cohesion is associated with perceived stress and depression, as well as lower life satisfaction. Stress emerged as a significant mechanism of mediating between family cohesion and digital self-regulation, suggesting that stress influences digital engagement. Family cohesion showed stronger relationships with emotional outcomes than direct regulation of digital behaviors.

**Discussion:**

By modeling these variables using SEM, this study demonstrates how family relationships shape digital well-being. Institutional ethical approval was obtained, and informed consent was provided by all participants.

## Introduction

Digitalization has profoundly transformed how individuals particularly adolescents and young adults, including university students connect, communicate, and cope with daily challenges. While digital tools offer opportunities in education, socialization, and productivity, their excessive use can increase stress and reduce psychological well-being (Newport, 2019; Twenge and Campbell, 2018). Families play a critical role in shaping digital habits and protecting young people from harmful use (Roman et al., 2025). In this context, digital self-regulation emphasizes simplicity and goal-oriented, conscious technology use (Newport, 2019). This study is grounded in Family Systems Theory, which posits that patterns of family interaction influence individual coping behaviors and self-regulation skills. This perspective provides a useful framework for understanding and regulating digital self-regulation among young adults.

Research highlights the importance of family cohesion in shaping both emotional well-being and digital behaviors. A supportive and harmonious family environment reduces stress and depressive states while enhancing life satisfaction (Bian et al., 2024). Conversely, stress can increase digital tool use as a temporary coping mechanism (Tadpatrikar et al., 2021). Weak family cohesion may lead individuals to seek temporary psychological relief through digital tools, heightening the risk of digital addiction (Reinecke and Hofmann, 2016). This weak relationship particularly affects young people and university students who remain emotionally connected with their families while managing their increasing digital autonomy (Zhu et al., 2022). Therefore, the interaction between family cohesion, stress, and digital self-regulation is critical for fostering healthy digital habits. Although previous studies have examined family harmony, stress, and unbalanced digital use separately, there are few studies that investigate how these variables interact to explain the development of healthy digital self-regulation habits among young adults. In particular, the mediating role of stress has not been examined in these studies.

By examining the mediating role of stress, this study contributes to the literature on family cohesion and digital habits within the context of young people, particularly young adults. Accordingly, the guiding research question is: does stress mediate the relationship between family cohesion and digital self-regulation among young adults? This study contributes to the field by theoretically expanding family systems theory to include digital self-regulation, methodologically testing a mediating model, and practically offering recommendations for family-based digital literacy and stress management methods. The findings indicate that interventions should focus on strengthening family cohesion, promoting open communication, and developing and scaling family-based digital literacy programs that are relevant to the realities and experiences of young people. For policymakers, awareness campaigns are recommended that emphasize conscious digital use and the protective role of family cohesion during emerging adulthood, when digital autonomy expands most rapidly. Furthermore, psychologists and educators are encouraged to develop new strategies for stress reduction beyond digital tools in order to foster, promote, and disseminate healthier digital habits and to support the well-being of young people as they navigate this transitional developmental period.

## Theoretical background

The family systems theory developed by Bowen (1978) and Kerr (2000) suggests that interactions among family members shape both problematic and healthy behavioral patterns. This theory provides a framework for fostering the emotional development of family members and reducing negative emotions such as stress and sadness (Johnson and Ray, 2016) which is especially relevant for young adults navigating emerging developmental responsibilities.

Establishing healthy communication within the family is of fundamental importance for psychological and social harmony. Open and honest communication promotes coherence in the thoughts and behaviors of family members (Lloyd et al., 2023). Families that maintain such communication development harmony in thoughts and behaviors. Enough and good family cohesion allows individuals to trust each other for emotional support and to meet their sensitive needs (Erkoç et al., 2021), a supportive mechanism that remains influential even as young adults become increasingly autonomous. Through this harmony, individuals can meet their emotional needs and free themselves from digital addictions, particularly among young adults who often turn to digital platforms for stress relief. In cohesive families, clearly defined boundaries regarding social media and digital technology use help maintain balance (Johnson and Francis, 2022), providing developmental scaffolding as young adults form independent digital habits. Indeed, family stress, crises, and problems may increase engagement with the digital world (Tadpatrikar et al., 2021). A pattern often observed during the emotionally sensitive phase of emerging adulthood (Park, 2020).

While family cohesion refers to the structural emotional bonding among family members, in this study, family harmony is considered a core element and a functional manifestation of this cohesion (Süzen Keşan et al., 2025). In this context, strong family cohesion serves as a protective factor in achieving digital self-regulation (Peleg and Peleg, 2024), creating a foundation from which young adults may learn and internalize healthy digital routines. Healthy communication, role modeling, boundary-setting, and stress reduction align with the core principles of digital self-regulation. While concepts such as digital detox and digital reduction emphasize temporary withdrawal from technology, the digital well-being literature increasingly highlights self-regulation and balance rather than abstinence. From this perspective, digital self-regulation represents a psychologically grounded approach that focuses on individuals’ capacity to manage digital engagement in line with their well-being needs (Vanden Abeele, 2021; Syvertsen and Enli, 2019). Family cohesion strengthens psychological and social well-being, supporting balance between digital and real life (Qi et al., 2024), a balance often challenged during the transitional period of emerging adulthood (Arnett, 2015). The family systems theory thus provides an appropriate framework for examining the impact of family cohesion on digital self-regulation and life satisfaction, particularly in the lives of young adults.

## Literature review related to hypotheses

The relationships between the variables specified in the study’s hypotheses were evaluated in the context of the existing literature and presented under relevant headings.

### Depression, family cohesion, and life satisfaction

Family cohesion refers to a structure in which family members have strong bonds with each other, common actions are stable, and healthy communication is established (Olson, 2000). Research shows that weak communication and low social support negatively shows concurrent patterns with family cohesion, leading to emotional problems in individuals (Fosco and Lydon-Staley, 2020).

Comprehensive studies have shown that there is a significant negative relationship between family cohesion and depression. Individuals who have achieved family cohesion have been found to have increased psychological resilience and reduced effects of negative experiences (Bian et al., 2024). As family cohesion increases, individuals’ ability to cope with stress also improves, while their depressive states decrease (Huang et al., 2022).

Depression is a mental state that negatively shows concurrent patterns with a person’s life, defined by symptoms such as sadness, hopelessness and social isolation that persist for most of the day (Yuping et al., 2024). Factors that increase the risk of developing depression include inadequate family communication, lack of social support, and weak family bonds (Chen and Harris, 2019).

Life satisfaction is an individual’s personal assessment of their quality of life. In families with high family cohesion, individuals’ life satisfaction is commonly reported as significantly higher (Ni et al., 2021). Similarly, systematic studies conducted with adolescents and children show a positive relationship between family cohesion and individuals’ perceptions of happiness and life satisfaction (Izzo et al., 2022). In summary, family cohesion increases an individual’s psychological resilience, reduces the risk of depression, and increases life satisfaction. In this regard, some hypotheses are proposed based on the literature. These findings are also meaningful for young adults, who, despite becoming more independent, often continue to rely on their families for emotional support and a sense of belonging.

### Hypotheses

*H1: There is a negative relationship between family cohesion and depression*.

There is a negative relationship between depression and family cohesion. The development of a healthy family environment is possible thanks to family cohesion, which supports open communication among family members (Olson, 2000). In a family unit, coping with stress, communication skills, the development of problem-solving abilities, and the presence of a sense of unity and belonging contribute to individuals viewing their lives from a more positive perspective (Bansal and Teotia, 2024). Individuals’ personal perceptions of their lives are a personal assessment that shows concurrent patterns with their level of life satisfaction. Life satisfaction can be defined as an individual’s satisfaction with their family, environment, and personal life (Diener et al., 1985). The most fundamental factors shows concurrent patterns within life satisfaction are social relationships, family dynamics, socialization, the feeling of being understood, helping others, and happiness (Ryff and Keyes, 1995).

One of the most comprehensive approaches explaining these factors is the PERMA Model developed by Seligman (2011). It is a valuable model in the field of positive psychology that assesses individuals’ happiness through positive emotions, engagement, relationships, life meaning, values, and the concept of success. According to the model, receiving sustainable support from family members and experiencing reciprocity in love and respect are among the fundamental elements that most increase life satisfaction (Carr, 2011). Indeed, a meaningful relationship has been observed between interpersonal communication and life satisfaction (Walsh, 2016). In this context, a family atmosphere where communication is open and supportive is often associated with higher levels of life satisfaction. This connection also makes sense for young adults, who may still draw emotional strength from their families even as they become more independent. Based on this body of work, an additional hypothesis is included in the present study.

*H2: There is a positive relationship between family cohesion and life satisfaction.*

Depression is a general mental disorder that negatively shows concurrent patterns with an individual’s cognitive functions and emotional state. Research indicates that people with depression experience impairment in functions such as decision-making, concentration, focus, memory, and mental flexibility (Gotlib, 2010; Dobielska et al., 2022).

People experiencing depression often view environmental situations negatively and perceive their lives as threatening and hopeless. With this perspective, they also form pessimistic expectations for the future (Everaert et al., 2017).

Furthermore, the negative effects of depression on life satisfaction are frequently mentioned in many studies. For example, it has been found that depressive behaviors are more common in individuals with low life satisfaction. An inverse relationship between depression and life satisfaction has been established (Joshanloo and Blasco-Belled, 2023). Research with young adult populations also points to a similar pattern: when family relationships are supportive and cohesive, young people tend to report feeling more satisfied with their lives and less overwhelmed by psychological strain. This suggests that, even as young adults move toward independence, family remains an important context in their overall well-being (Morales Almeida et al., 2024). Based on these data, the hypothesis that depression reduces life satisfaction is grounded on a logical basis.

*H3: There is a negative relationship between digital self-regulation and stress.*

Digital self-regulation is a capacity that encourages individuals to use digital technologies consciously, selectively, and in a controlled manner (Newport, 2019). This approach facilitates time management, reduces distractions, and strengthens focus. Research shows that digital self-regulation are associated with lower screen time, lightens individuals’ cognitive load, and increases their psychological well-being (Waseem et al., 2024). Excessive exposure to digital devices increases stress levels in adults, and especially among young adults; whereas more regulated and mindful patterns of digital use are linked to lower perceived stress (Twenge and Campbell, 2018; Saleem and Jan, 2024). Within this framework, a negative relationship between digital self-regulation and stress has been noted.

### Family cohesion, stress, and digital self-regulation

Family cohesion can be defined as the ability of individuals to act together and adapt when their families face difficulties (Walsh, 2016). The ability of individuals to manage stress is directly shows concurrent patterns with by the level of family cohesion. A supportive, protective, and harmonious family environment helps its members become more resilient emotionally and psychologically and has a protective effect against the stressors they encounter. Open communication and cooperation within the family ensure that stressful situations are resolved more effectively and easily (Bowen, 1978).

In today’s digital age, the constant state of being online and the endless flow of information have become a new source of stress in the lives of individuals and families. Digital self-regulation, which has emerged as a solution to this situation, is a philosophy of using technology consciously, mindfully, and based on need (Newport, 2019). Those who embrace this approach aim to achieve a calm and peaceful mind by choosing digital tools based on whether they add value to their lives and avoiding distracting and unnecessary tools (Odell, 2019). In this way, they can distance themselves from the artificial stress that digital tools create in their minds and focus more on real activities and interactions.

*H4: Depression is linked to the relationship between family cohesion and life satisfaction.*

Family cohesion and digital self-regulation are two powerful strategies that complement each other, enabling individuals to manage the stress they experience in the digital age. Families who practice digital self-regulation find opportunities to spend more quality time together without being distracted by technology’s disruptive effects on attention and focus. For example, as a digital self-regulation activity, they encourage face-to-face communication and deep conversations within the family by staying away from phones during dinner. Such joint digital detox practices strengthen family bonds and build a happier and healthier family environment by reducing individual stress (Uluçay and Kobak, 2020). Finally, it shows that digital detox, staying away from phones and other digital devices, can help people feel emotionally lighter and connect more with others. This digital detox within the family environment can help identify problems, create more space for conversation and closeness, and ultimately strengthen the family’s overall dynamics (Kolhe and Naik, 2025). In conclusion, digital self-regulation strengthens family cohesion and supports a family environment with lower stress levels and stronger family bonds.

*H5: There is a positive relationship between Family Cohesion and Digital self-regulation.*

Stress is a psychological pressure that arises as a result of the difficult imbalances individuals experience between environmental demands and their own internal states (Conger et al., 1999). Research shows that high stress levels negatively shows concurrent patterns with individuals’ physical and psychological health and weaken their problem-solving and decision-making abilities (Sarmiento and Arkadaşları, 2024). Increases in stress levels have been shown to weaken family communication, create emotional distance, and cause disintegration effects in family dynamics (Falconier, 2023). Recent work with young adults suggests that when family relationships feel supportive and close, young people tend to handle stress more effectively and report feeling better overall. These findings emphasize that family still matters in how young adults respond to stressful experiences (Morales Almeida et al., 2024). In inividuals with strong family communication, the negative effects of stress are reduced, stress coping skills develop with the emotional support provided, and individuals become more psychologically resilient (Zeng, 2021). Family members who share their stressful situations with each other experience a reduction in their burdens and are able to provide emotional support to each other (Moreira and Telzer, 2015). In individuals whose family structures are based on communication rooted in trust, acceptance, and love, psychological harm is reduced during crisis situations, and balance is achieved at the neurobiological level, offering the individual an opportunity for both mental and physical empowerment (Larson, 2024). For example, a longitudinal study conducted with some families during the COVID-19 pandemic showed that high family cohesion reduced the negative relationship between economic hardship and parental depression (Wilkinson-Lee et al., 2025).

In conclusion, individuals with high family cohesion are better able to cope with stressful situations. Based on the literature, our hypothesis is as follows:

*H6: There is a negative relationship between family cohesion and stress.*

Some studies show that excessive use of digital technologies can increase stress and anxiety levels in individuals (Kuss and Griffiths, 2017). As stress levels increase, individuals tend to spend more time in digital environments and turn to more content, which also increases the likelihood of individuals becoming digitally addicted (Montag et al., 2020). Individuals use digital environments as a method of coping with stress (Kardefelt-Winther, 2014). Patterns of increased digital addiction have been shown to co-occur with lower psychological well-being and may also be accompanied by difficulties in family relationships, including weakened communication and relational strain (Coyne et al., 2021; McDaniel and Coyne, 2016). High family cohesion can reduce the effects of digital addiction and serve as a protective factor for individuals. Strong communication, sharing, and support within the family, including during young adulthood when digital interaction intensifies but family ties remain meaningful, makes it easier for individuals to cope with stress (Arnett and Schwab, 2012). To further clarify this dynamic, it is important to examine whether stress acts not only as a direct factor but also as a mechanism linking family cohesion and digital self-regulation.

**Figure 1:**
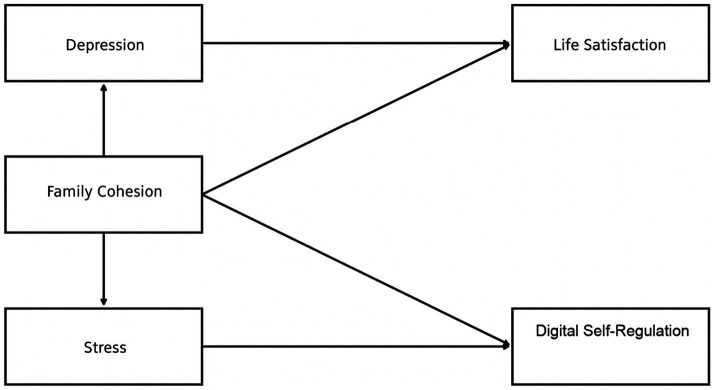
Research model.

The research model, which was developed based on the hypotheses to be tested within the scope of the study, is presented in Figure 1.

*H7: Stress is linked to the Relationship between Family Cohesion and Digital self-regulation.*

Digital self-regulation is linked to reduced individuals’ dependence on digital technologies, supports quality time within the family, and encourages conscious and selective use of digital tools (Newport, 2019; Waseem et al., 2024). Therefore, stress appears to be an important factor in the relationship between digital self-regulation and family cohesion in the context of digital addiction, including among young adults, for whom digital engagement often intensifies while family ties remain meaningful.

## Materials and methods

This section may be divided by subheadings. It should provide a concise and precise description of the experimental results, their interpretation, as well as the experimental conclusions that can be drawn.

### Study design, participants, and procedures

This study was designed as a cross-sectional study. Convenience sampling was used to reach participants, and data were collected via an online survey (Leavy, 2017). The study population consisted of young adult university students studying in Turkey. The minimum sample size required for a heterogeneous distribution at a 95% confidence interval was calculated to be 384 (Taherdoost, 2018). Accordingly, a total of 406 volunteer students who voluntarily agreed to participate constituted the research sample. The online survey link was disseminated through the researchers’ academic and social networks and further shared among participants, reflecting a convenience-based and snowball-like sampling approach. This sampling strategy was deemed appropriate for reaching a representative sample of young adult college students in line with the study’s objectives; however, the non-probability nature of the sample may limit the generalizability of the findings.

The data collection process was conducted in September 2025. Participants were provided with an informed consent form explaining the purpose of the study, the scales used, and the confidentiality of personal data, and their voluntary consent was obtained. The Family Harmony Scale, Stress Scale, Depression Scale, Life Satisfaction Scale, and Digital Minimalism Scale were used in the study. Since the Digital Minimalism Scale was not available through open access, it was requested by emailing the owner of the questionnaire. The other scales were accessed through open access. Ethical approval for this study was obtained from the Ethics Committee of Üsküdar University (Decision No: 61351342/020-1,416, Date: 27.08.2025). The approval was obtained from Üsküdar University in order to obtain the ethics report more quickly, as the author’s affiliated university was on summer break at the time of application.

### Measures

In addition to identifying participants’ sociodemographic characteristics in the personal information form, four different scales were used in the study. The PLS-SEM approach was preferred in this study because the primary objective is to examine the explanatory and predictive power of psychosocial variables on digital self-regulation, rather than validating causal relationships within the framework of strict fit criteria. Furthermore, the presence of high correlations between variables and the exploratory nature of the model rendered the variance-based SEM approach more appropriate. All of the scales used in the study have previously been validated in both their original versions and as Turkish adaptations; for the sake of transparency, the reliability coefficients obtained from the current sample are presented below.

### Family cohesion scale

The scale, originally developed by Kavikondala et al. (2016), was adapted into Turkish by Duman Kula et al. (2018). The scale consists of five items and aims to assess participants’ self-reported levels of family cohesion. The scale uses a 5-point Likert-type rating (1 = “strongly disagree” – 5 = “strongly agree”). The Turkish adaptation study confirmed the scale’s unidimensional structure and high internal consistency coefficient (*α* = 0.92) (Kula et al., 2018). In this study, the Cronbach’s Alpha reliability coefficient of the scale was found to be 0.941, indicating high internal consistency. In this study, family harmony is considered a core element of family cohesion.

### DASS-21 Scale

The Depression, Anxiety, and Stress Scale (DASS-21) was originally developed by Lovibond and Lovibond (1995). The scale consists of 21 items and three subscales (depression, anxiety, stress). The Turkish adaptation was conducted by Yılmaz et al. (2017), and the psychometric properties of the scale were shown to be valid and reliable in Turkish culture. In the Turkish adaptation study, the reliability coefficients of the scale were reported as *α* = 0.89 for depression, *α* = 0.819 for anxiety, and *α* = 0.755 for stress (Yılmaz et al., 2017). In this study, the depression and stress dimensions were used, and *α* = 0.985 for the depression dimension rated on a 5-point Likert scale and *α* = 0.818 for stress were found, indicating a very high internal consistency.

### Life Satisfaction Scale

The Life Satisfaction Scale was developed by Diener et al. (1985). The scale consists of five items designed to assess individuals’ life satisfaction. It was adapted into Turkish by Köker (1991) and later revised by Dağlı and Baysal (2016). The Turkish version has a high internal consistency coefficient (*α* = 0.88) (Dağlı and Baysal, 2016). In this study, the Cronbach’s Alpha reliability coefficient of the 5-point Likert-type scale was found to be 0.894.

### Digital self-regulation (measured via Digital Minimalism Scale)

Digital self-regulation in this study was operationalized using the Digital Minimalism Scale developed by Waseem et al. (2024). The scale consists of nine items assessing individuals’ tendency to consciously limit, structure, and manage their digital media use. Although the original scale is named “digital minimalism,” in this study the construct is conceptually framed within the broader literature on digital self-regulation, which refers to the intentional capacity of young people to monitor, restrict or modulate digital engagement. Accordingly, digital minimalism is treated as one behavioral expression of digital self-regulation rather than an isolated theoretical construct.

Internal reliability in the present sample was high (*α* = 0.875 for Digital Intent; *α* = 0.910 for Digital Detox), based on a 5-point Likert-type response format. In this study, the reliability coefficients of the 5-point Likert-type Digital self-regulation scale were found to be *α* = 0.875 for Digital Intent and *α* = 0.910 for Digital Detox, indicating a high level of internal consistency.

### Data collection

Ethical approval was obtained prior to data collection for the study. Since the author’s own university ethics committee was in recess during the summer break, the approval was received from another university to ensure the timely initiation of the study. After receiving approval, the survey form was transferred to the Google Forms system. During this process, participants were informed about the study’s objectives, methodology, and confidentiality. An informed consent form was obtained electronically from all participants. They were given the freedom to withdraw from the survey at any stage. The data collection process took place between September 1 and September 20. During this period, surveys that were incomplete were excluded from the analysis. The surveys were collected through random sampling by reaching out to individuals in the researchers’ circles, aiming to achieve a sufficiently representative sample. The survey was closed when 406 responses were received. No minors (under the age of 18) were included in the study. “In line with the revised conceptual framework of this manuscript, digital self-regulation is examined descriptively rather than causally, and the scale scores are used to map associations rather than to test predictive effects.”

### Statistical analyses

The data obtained in the study were analyzed using SPSS 25.0 and SmartPLS software packages. A multistage analysis process was followed to test the hypotheses formulated in the study. In the first stage, reliability analyses and exploratory factor analysis (EFA) were performed in the SPSS 25.0 environment to determine the internal consistency of the scales used. In the second stage, frequency analyses were performed to identify the demographic and social media usage characteristics of the participants. Subsequently, correlation analyses were used to examine the relationships between the variables included in the study. In the final stage, confirmatory factor analysis (CFA) and structural equation modeling (SEM) were applied using SmartPLS software to test the validity of the proposed structural model with-in the scope of the research. Prior to parametric analyses, relevant statistical assumptions, including normality, were examined and found to be acceptable. After all analyses were completed, the findings were systematically reported in tables.

### Findings

#### Participant characteristics

In this section of the study, the demographic characteristics of the participants are presented. As seen in Table 1, the characteristics of the participants are as follows:

**Table 1:** Demographic characteristics of participants.

Variable	Category	*N*	%
Gender	Female	203	50.0
	Male	203	50.0
Frequency of social media use	1–3 times a day	14	3.4
	3–5 times a day	143	35.2
	More than 5 times a day	249	61.3
Social media usage time	1–3 h	9	2.2
	3–5 h	157	38.7
	More than 5 h	240	59.1
What is the most widely used social media platform? Average age: 20.48	Facebook	211	52.0
	Instagram	386	95.1
	Twitter/X	347	85.5
	TikTok	210	51.7
	Snapchat	184	45.3
	LinkedIn	39	9.6
	YouTube	398	98.0

As shown in Table 1, the gender distribution of the 406 participants is balanced (50% female, 50% male), indicating a homogeneous sample. The majority are young adults, with the average age of 20.48 and most participants in the 20–25 age range (54.0%). All participants are students.

Regarding social media use, most participants use it more than five times a day (61.3%) and spend over 5 h daily (59.1%), reflecting the digital generation’s intensive engagement. Instagram (95.1%) and YouTube (98.0%) are the most popular platforms, while TikTok (51.7%) also shows high usage among young users, consistent with recent research (Montag et al., 2021). Participants mainly use social media for entertainment (97.3%) and maintaining social relationships (92.6%).

#### Correlations among variables

When examining the results of the correlation analysis, significant relationships were found between the variables (Table 2). First, a positive and high level relationship was found between Family harmony and Life Satisfaction (*r* = 0.847, *p* < 0.01). This finding shows that family relationships have a critical impact on individuals’ overall satisfaction with life.

**Table 2:** Correlations between variables.

	Mn.	Sd.	1	2	3	4	5
1. FH	3.84	0.83	1				
2. LS	3.86	0.90	0.847**	1			
3. DM	2.21	0.57	−0.642**	−0.679**	1		
4. STR	2.18	0.53	−0.496**	−0.476**	0.906**	1	
5. DPR	2.18	0.60	−0.427**	−0.410**	0.843**	0.942**	1

FH, family harmony; LS, life satisfaction; DM, digital minimalism; STR. stress; DPR, depression; Mn, mean, Sd., standard deviation.

**p < 0.0.1, *p < 0.0.5.

As seen in Table 2, in contrast, negative correlations were found between Family harmony and Digital self-regulation (*r* = −0.642, *p* < 0.01), Stress (*r* = −0.496, *p* < 0.01), and Depression (*r* = −0.427, *p* < 0.01). These results reveal that as family cohesion increases, individuals’ tendency toward digital self-regulation decreases, while stress and depression levels also decline.

Similarly, significant negative correlations were found between Life Satisfaction and Digital self-regulation (*r* = −0.679, *p* < 0.01), Stress (*r* = −0.476, *p* < 0.01), and Depression (*r* = −0.410, *p* < 0.01). This indicates that individuals with high life satisfaction experience less stress and depression and show a lower tendency toward digital self-regulation.

On the other hand, positive and highly significant correlations were found between Digital minimalism and Stress (*r* = 0.906, *p* < 0.01) and Depression (*r* = 0.843, *p* < 0.01). This finding suggests that individuals who embrace digital self-regulation may have higher levels of stress and depression. Finally, a very high positive relationship (*r* = 0.942, *p* < 0.01) was found between Stress and Depression, indicating that psychological problems co-occur, as expected.

Overall, the findings reveal that family cohesion and life satisfaction are protective factors, while stress and depression are risk factors. Furthermore, the strong relationships between digital self-regulation and psychological variables indicate that this construct may be a critical variable for individuals’ mental well-being.

Before conducting the confirmatory factor analysis (CFA), correlations among the scale items were examined, and several highly correlated items were removed from the model. According to Hair et al. (2019), strong inter item correlations indicate redundancy and may lead to multicollinearity, while Kline (2015) notes that such overlap can weaken model validity. Therefore, a limited number of items were excluded to enhance measurement reliability without compromising conceptual integrity. The removed items were FH5 (Family Harmony), YM4 (Life Satisfaction), DN2, DN5, DS7 and DS9 (Digital Minimalism), STR5–STR7 (Stress), and DPR3, DPR4, DPR6 (Depression) (Table 3). Following these adjustments, the final measurement model was established, and CFA was performed.

**Table 3:** Confirmatory factor analysis results.

	AVE	CR	Load.	*t* value	VIF
FH (Cronbach’s *α* = 0.647)	0.733	0.710			
FH 1			0.911	132.766	1.296
FH 2			0.798	20,452	1,296
LS (Cronbach’s *α* = 0.964)	0.966	0.965			
LS 1			0.983	245.402	7,575
LS 5			0.982	223,680	7,515
DM (Cronbach’s *α* = 0.881)	0.668	0.936			
DM1			0.813	21.792	2.192
DM3			0.794	47,947	1,477
DM4			0.904	36,942	5,483
DM6			0.803	17,664	2,787
DM8			0.764	10,204	2,419
STR (Cronbach’s *α* = 0.917)	0.797	0.944			
STR1			0.890	58.593	2.505
STR2			0.913	32,308	6,149
STR3			0.868	23,061	4,758
STR4			0.900	55,458	2,988
DPR (Cronbach’s *α* = 0.930)	0.876	0.944			
DPR2			0.908	51.664	2,682
DPR5			0.949	75,280	5,683
DPR6			0.951	43,044	6,420

FH, family harmony; LS, life satisfaction; DM, digital self-regulation; STR, stress; DPR, depression; AVE, average variance explained; CR, composite reliability; LOAD, factor load; *t* value, *t*-Value; VIF, variance inflation factor.

During confirmatory factor analysis (CFA), several items (FH3, FH4, YM2, YM3, DPR1) were removed to enhance reliability and validity (Table 3). Hair et al. (2019) recommend excluding items with loadings below 0.70, especially when they reduce overall reliability. Similarly, Kline (2016) notes that low loadings or high error covariances can weaken model fit, while Fornell and Larcker (1981) emphasize that indicators must load sufficiently to ensure validity. In this study, the excluded items had low loadings, high VIF values, and reduced discriminant validity. As noted in the literature, such exclusions are acceptable when theoretical integrity is preserved and model robustness is improved (Brown, 2015; Hair et al., 2019). Although certain items were excluded from the measurement model for statistical reasons, the items retained in the final model consist of indicators that represent the theoretical core of the respective constructs. Nevertheless, as the reduction in the number of items may limit content validity, the findings have been interpreted with caution.

Only items with high factor loadings, acceptable VIF values, and sufficient validity and reliability were retained in the final measurement model, consistent with the recommendation that “measurement models should be robust not only statistically but also conceptually” (Henseler et al., 2015, p. 125).

The validity and reliability of the study’s scales were tested through confirmatory factor analysis (CFA). Hair et al. (2019) note that CFA assesses construct validity across three dimensions: convergent validity, discriminant validity, and internal consistency reliability.

For internal consistency, Cronbach’s Alpha and Composite Reliability (CR) values were examined. Nunnally (1978) suggests *α* > 0.70 as acceptable. In this study, Cronbach’s Alpha values were 0.647 for Family Harmony, 0.964 for Life Satisfaction, 0.881 for Digital self-regulation, 0.917 for Stress, and 0.930 for Depression. While the Family cohesion subscale is slightly below the threshold, all other scales show strong internal consistency. The decrease in the Cronbach’s Alpha coefficient reported for the Family Harmony Scale stems from the difference between utilizing all items during the exploratory analysis phase and retaining only those with high factor loadings during the confirmatory analysis process. Given that Cronbach’s Alpha is a measure sensitive to the number of items, a decline following item reduction is an expected outcome. Therefore, internal consistency during the confirmatory analysis phase was evaluated based on Composite Reliability (CR) and Average Variance Extracted (AVE) values. Although the Life Satisfaction and Family Cohesion constructs were represented by two items in the confirmatory process, the exceptionally high factor loadings and AVE/CR values of these items support the measurement reliability. Additionally, all CR values above 0.70 confirm adequate composite reliability (Fornell and Larcker, 1981).

Convergent validity: Factor loadings and Average Variance Extracted (AVE) values were examined. Fornell and Larcker (1981) suggest AVE > 0.50, which was achieved in this study with values of 0.733 (Family Harmony), 0.966 (Life Satisfaction), 0.668 (Digital self-regulation), 0.797 (Stress), and 0.876 (Depression). These results confirm convergent validity for all constructs. Hair et al. (2019) also recommend item loadings above 0.70; in this study, loadings ranged from 0.764 to 0.983, meeting this criterion.

Multicollinearity (VIF): VIF values were examined in the CFA results. According to Kock (2015), values below 3.3 indicate no common method variance issue. Although some items exceeded 5, overall VIF values were acceptable. As Hair et al. (2019) note, values below 10 do not indicate a statistical concern.

#### Discriminant validity

To assess whether the study scales were distinct, Heterotrait-Monotrait Ratio (HTMT) values were examined (Henseler et al., 2015). According to the literature, HTMT values below 0.85 indicate strong, and values below 0.90 indicate acceptable discriminant validity (Henseler et al., 2015; Franke and Sarstedt, 2019).

Table 4 shows that some HTMT values (e.g., FH–LS = 1.088; DM–DPR = 0.958; STR–DM = 1.029) exceed the threshold, indicating conceptual overlap and limited discriminant validity. The strong correlation between Family Harmony and Life Satisfaction suggests these constructs are not fully distinct, aligning with Fornell and Larcker’s (1981) view that overlapping concepts require careful modeling.

**Table 4:** discriminant validity results.

	FH	DPR	DM	STR
FH				
DPR	0.550			
DM	0.802	0.958		
STR	0.648	1.021	1.029	
LS	1.088	0.433	0.681	0.508

FH, family harmony; LS, life satisfaction; DM, digital self-regulation; STR, stress; DPR, depression.

However, exceeding the 0.90 HTMT threshold does not necessarily invalidate the model. As Henseler et al. (2015) note, such cases may occur in social sciences for conceptually related variables and should be interpreted within the theoretical context. Thus, although discriminant validity is somewhat limited, the findings remain meaningful within the study’s framework.

#### Model fit

The standardized root mean square residual (SRMR) of the saturated model is 0.127, exceeding the recommended threshold of 0.08 (Hu and Bentler, 1999). However, recent studies emphasize that model fit indices in PLS-SEM should be interpreted with caution. Hair et al. (2019) note that global fit measures like SRMR or NFI are secondary since PLS-SEM focuses on prediction, not covariance. Similarly, Henseler et al. (2016) state that SRMR provides supportive information, but the main focus should be on reliability, validity, and predictive accuracy (e.g., AVE, CR, *R*^2^, *Q*^2^). Therefore, this study prioritizes measurement validity and the predictive power of the structural model over traditional fit indices. Furthermore, the high SRMR value indicates the presence of conceptual overlaps within the measurement model and suggests that the results should be evaluated within an explanatory framework rather than with confirmatory precision.

#### Direct and indirect effects

As shown in Table 5, family harmony has a negative and significant effect on depression (*β* = −0.429, *t* = 6.513, *p* < 0.001, *f*^2^ = 0.226) and a strong positive effect on life satisfaction (*β* = 0.889, *t* = 61.439, *p* < 0.001, *f*^2^ = 3.546). Digital self-regulation negatively and significantly shows concurrent patterns with stress (*β* = −0.502, *t* = 8.001, *p* < 0.001, *f*^2^ = 0.337).

**Table 5:** Hypothesis tests.

H	Path	Std. *ß*	Std. error	*t* value	*p*	*f* ^2^	Confidence interval	
							%2.5	97.5
Direct hypotheses								
H1	FH → DPR	−0.429	0.066	6.513	0.000	0.226	−0.556	−0.300
H2	FH → DM	−0.160	0.051	3.158	0.002	0.193	−0.256	−0.062
H3	FH → STR	−0.502	0.063	8.001	0.000	0.337	−0.611	−0.367
H4	FH → LS	0.889	0.014	61.439	0.000	3.546	0.859	0.914
H5	DPR → LS	−0.035	0.024	1.442	0.149	0.005	−0.083	0.012
Mediation hypotheses								
H6	FH → DPR → LS	0.015	0.011	1.353	0.176		−0.005	0.038
H7	AFH → STR → DM	−0.431	0.057	7.519	0.000		−0.540	−0.316

FH, family harmony; LS, life satisfaction; DM, digital self-regulation; STR, stress; DPR, depression; H, hypothesis; Path, path coefficient; Std. *β*, standardized beta coefficient; Std. Error, standard error; *t* value, *t*-Value; *p*, significance level; *f*^2^, effect size; confidence int., confidence interval.

Associational pathways linking depression between family cohesion and life satisfaction was not significant (*β* = 0.015, *t* = 1.353, *p* > 0.05). Contrary to expectations, the relationship between family cohesion and digital self-regulation was negative (*β* = −0.160, *t* = 3.158, *p* < 0.01, *f*^2^ = 0.193). Finally, family harmony also had a negative and significant effect on stress (*β* = −0.502, *t* = 8.001, *p* < 0.001, *f*^2^ = 0.337), and stress played a significant mediating role between family harmony and digital self-regulation (*β* = −0.431, *t* = 7.519, *p* < 0.001). While the mediation effect of stress between family cohesion and digital self-regulation is statistically significant, this result should be considered exploratory due to the substantial conceptual overlap among stress, digital self-regulation, and depression.

## Discussion and conclusion

According to the results of our study’s findings, most of the seven hypotheses were significantly supported. However, two important findings, namely the lack of support for H4 and the contradictory result of H5, require further consideration and discussion within the context of the study sample and design. In interpreting these findings, both statistical significance and estimates of effect size were taken into account to ensure a more balanced assessment of the observed relationships.

The study found a negative and significant relationship between family cohesion (FH) and depression (DPR) (H1), consistent with prior research showing that a supportive family environment is associated with lower levels of depressive symptoms (Eisman et al., 2015; Ngai and Ngu, 2013). Healthy communication and mutual understanding within families may enhance emotional regulation and may be linked to lower depression risk (Izzo et al., 2022). As the closest social unit, the family is widely discussed as an important context for the development of psychological resilience and coping capacities (Uruk et al., 2007). Other studies similarly suggest that individuals in harmonious families manage daily stress more effectively, which is linked with strengthening resilience and mitigating depressive symptoms (Falconier, 2023). Overall, a supportive and secure family environment appears to be associated with healthier coping patterns and emotional strength, helping individuals handle external difficulties more easily.

Another key finding is the positive relationship between family cohesion and life satisfaction (H2). Prior research shows that family cohesion is positively associated with higher life satisfaction, lower feelings of loneliness, and supports personal well-being (Roman et al., 2025; Rahal and Fosco, 2025). Individuals with strong family cohesion report greater enjoyment and a more positive outlook on life (Qin et al., 2015). These associations remain meaningful for young adults, who often navigate increasing independence while continuing to rely on family relationships as an important emotional resource. Thus in this regard, maintaining family cohesion is closely linked with higher levels of life satisfaction.

Another key finding is the negative relationship between digital self-regulation and stress. Previous studies show that reduced focus problems and fewer distractions are associated with lower stress levels (Twenge and Campbell, 2018). Excessive digital exposure has been associated with concentration difficulties, whereas digital self-regulation is often linked with improved focus and psychological well-being through lower perceived stress.

Associations among depression in the relationship between family cohesion and life satisfaction (H4) was not supported. Although depression is widely recognized as a risk factor related to lower life satisfaction, in this study the strong direct association between family cohesion and life satisfaction appeared to outweigh the expected indirect pathway through depression. Prior research indicates that emotional and social well-being, rather than depression, are more significant pathways to psychological health in cohesive families (An et al., 2024). The high impact of family cohesion on life satisfaction likely diminished the mediating role of depression, suggesting that other factors such as emotional and social well-being may play a greater role. Given depression’s cyclical nature, its effects may not have been fully visible in this study. Since family cohesion is a dominant variable that directly enhances life satisfaction, the effect of depression has diminished.

The hypothesis of a positive relationship between Family Cohesion and Digital self-regulation was not confirmed (H5). The literature emphasizes that individuals with high family cohesion tend to have healthier technology usage patterns. It is stated that strong family bonds are associated with reduced the use of digital devices (Coyne et al., 2021). Family support reduces stress and may co-occurs with the development of healthy technology usage habits. However, the inconsistency found in this study can be explained by several reasons. Individuals with high family cohesion may use the digital environment in a balanced way because they meet many of their emotional needs within the family. Digital self-regulation may be less of a priority for this group (Singh and Tyagi, 2023). This finding differs from some previous studies that reported a direct protective role of the family environment on digital habits. This suggests that family cohesion among young adults may exert its influence not through direct behavioral control, but rather through more indirect psychological effects, such as stress. Harmonious families who do not practice digital self-regulation may use technology for things like sharing, family communication, and socializing that support technology and family values and strengthen family belonging. Therefore, digital devices may be used mindfully in a way that positively shows concurrent patterns with family cohesion. Furthermore, this finding suggests the possibility of a two-way cycle. It is not entirely clear whether the digital world negatively shows concurrent patterns with family cohesion or whether family discord shows concurrent patterns with the frequency of digital world use.

Another finding of the study supports a negative relationship between family cohesion and stress (H6). It reveals that family cohesion and healthy communication patterns are associated with lower levels of perceived stress (Rivera et al., 2008). Families with strong family cohesion may provide a natural psychological safety net that enables individuals to cope with stressful situations. It is known that family members who always feel understood and supported are more resilient to stress arising outside the family (Roman et al., 2025). Family cohesion provides essential support in processes such as collaborative problem-solving and emotional sharing. Individuals raised in harmonious family environments built on trust, love, and respect demonstrate greater psychological flexibility compared to those from families characterized by conflict and dysfunctional communication. Family cohesion functions as a key protective factor that alleviates stress. Returning home to an atmosphere of peace and shows concurrent patterns within allows individuals to unwind and regain emotional balance, whereas exposure to conflict and tension at home can heighten stress levels even in the absence of external pressures. In this way, family unity functions as an important protective factor in stress-related processes for young adults and all family members.

The final finding of the study confirmed associational pathways linking stress on the relationship between family cohesion and digital self-regulation. Especially during young adulthood, when individuals continue to rely on family support while navigating increased digital engagement, weakening family bonds have been discussed as being linked to a greater search for external validation and social interaction through digital environments, weakening family bonds trigger a search for external validation and socialization, leading individuals to turn to the digital world to fulfil these needs (Twenge and Campbell, 2018). Individuals turn to the digital world as a coping mechanism for stress, but this provides only short-term relief. In this context, stress levels are lower in communities with high family cohesion, which facilitates the internalization and implementation of digital self-regulation (Reinecke and Hofmann, 2016). When stress levels are low, individuals can act in a controlled manner by using digital environments more consciously and in a needs-oriented way. Individuals experiencing conflict within the family may increase stress and digital use. However, increased digital use habits can also worsen family communication, making family cohesion more difficult. Screen time restrictions alone are not sufficient in this cycle; family problems must be resolved. Therefore, the mediating role of stress is seen as an important mechanism explaining the effect of family bonds on digital behavior.

While the study found that the relationship between family cohesion and digital self-regulation was not directly shows concurrent patterns with, the fact that stress is linked to the relationship between family cohesion and digital self-regulation seems contradictory. However, we assumed that individuals with high family cohesion would directly turn to digital self-regulation (Wei et al., 2025). Individuals with high family cohesion can maintain good family relationships without needing digital self-regulation. They can also use digital tools to strengthen family bonds. When stress is considered as an intervening factor, family cohesion is associated with lower stress levels, and reduced stress may be linked with lower reliance on digital tools as coping mechanisms (Hamdi Bacha and Benseghir, 2023). Thus, as seen, family cohesion does not directly show a statistically significant association with digital self-regulation but is indirectly related concurrent patterns with it through psychological effects such as stress. This conclusion shows us that digital self-regulation is practiced to prevent the use of the digital world, which is a psychological escape for individuals, as a tool. Individuals with good family cohesion do not feel the need to escape because the stress factor is low.

In summary, the findings of this study largely coincide with the existing literature. However, other factors associated with digital self-regulation should not be overlooked. Family cohesion is consistently associated with lower stress levels, and stress emerges as an important linking mechanism between family cohesion and digital self-regulation. It also suggests that confirms that individuals use digital tools, such as scrolling through screens and playing games, as a short-term method of coping with stress. This carries the risk of individuals becoming dependent on digital tools as a method of psychological relief over time. Digital self-regulation, which enables conscious use of digital tools by overcoming digital addictions, can support achieving balance in this regard. From a theoretical perspective, these findings demonstrate that family relationships remain relevant even during early adulthood, thereby extending Family Systems Theory to the realm of digital behavior. From a practical standpoint, the findings suggest that approaches focused solely on limiting screen time may only be effective if supported by family communication and stress management. These findings should be interpreted as associational patterns rather than causal effects, given the cross-sectional design of the study. Overall, the results indicate that family cohesion continues to play a meaningful role in shaping stress experiences and digital behaviors among young adults, despite the growing centrality of digital technologies in this stage of life.

### Study limitations

Performing both exploratory and confirmatory factor analyses on the same dataset may increase the risk of chance capitalization on fit. However, since the objective of this study was to examine the behavior of the scales within this specific sample, the analyses were conducted within an exploratory framework. This study are indicative of the relevance of a harmonious, healthy, empathetic, and supportive family environment in relation to mental health and increasing life satisfaction, particularly during young adulthood, a developmental period characterized by heightened emotional sensitivity and transitional life demands. The findings also suggest that the association between family cohesion and depression may reflect through shared psychological mechanisms, reflecting a deeper pattern of interconnected emotional processes rather than a simple relationship. Furthermore, the complex and bidirectional nature of the relationship between digital habits and family dynamics has been revealed. The findings suggest that difficulties in family and particularly among young adults communication were observed to co-occur with higher levels of digital technology use, and this pattern is also observed alongside weaker perceptions of family connectedness. At the same time, various other contextual and individual factors that could influence the relationships between family harmony, stress, and digital self-regulation, particularly among young adults, were not included in this study. Indeed, variables such as socioeconomic status, personal psychological state, peer relationships, etc., can affect both stress levels and digital behaviors in young adulthood. However, in line with the main objective of the study, the analyses focused on family processes as a central social context that continues to play a significant role in the increasing autonomy process in young adults. Future studies including these additional variables could contribute to the development of more comprehensive explanatory models. These patterns are particularly noteworthy given that the study sample consisted of young adults, a group for whom both family relationships and digital engagement remain salient.

### Synthesis of key findings

This study reaffirms the fundamental role that a harmonious, healthy, empathetic, and supportive family environment is associated with mental health and increasing life satisfaction. The study also indicate that the relationship between family cohesion and life satisfaction may involve shared psychological processes related to depression, pointing to a deeper underlying structure beyond a simple correlational pattern. Furthermore, the results highlight the complex and potentially bidirectional nature of the relationship between digital habits and family dynamics. Family communication difficulties were found to co-occur with higher levels of digital use, while increased digital engagement was also observed alongside weakened family bonds. Taken together, these patterns seem particularly relevant in the context of young adulthood, a life stage in which digital engagement intensifies while family relationships continue to function as an important emotional reference point.

### Contributions of the study to the literature

This study contributes to the literature by integrating the network of relationships between family harmony, digital habits, and stress, focusing on young adults. The findings support existing theoretical discussions by illustrating demonstrating the position of depression within these relationships. Importantly, the study emphasizes that the connections between the variables examined are more layered and context-dependent than they initially appear. By focusing on a sample of young adults in a specific cultural setting, the findings highlight the value of considering developmental and contextual factors in this area. Overall, these results encourage future research to adopt more sophisticated designs that are sensitive to cultural context and longitudinal dynamics.

### Recommendations for researchers

Based on the findings of this study, several important recommendations can be offered for future research.

(1) Conducting longitudinal studies would be beneficial for understanding the true causal relationship between digital tool usage and family dynamics. This type of design could help clarify the cause-and-effect relationship by tracking changes in variables over time.
(2) The study could be conducted on other age groups and cultural environments beyond those included in the current study to produce more generalizable research.
(3) To resolve the contradictory hypothesis in the study, future research may benefit from break down digital tool usage into more specific behaviors. For example, research could be useful that examines the difference between the effects of digital communication with family members and the effects of digital tool usage for purposes such as entertainment, relaxation, and leisure.
(4) Various awareness campaigns may be developed to address digital addictions.
(5) Digital self-regulation studies based on family cohesion could be expanded.
(6) The contributions of digital self-regulation to families and individuals’ life satisfaction may be promoted through public service announcements.
(7) Awareness initiatives may also focus on the relationship between digital addictions and stress factors.
(8) The importance of family cohesion in society could be emphasized, and its contribution to reducing psychological distress should be highlighted.
(9) Families may consider establishing a digital contract regarding digital self-regulation, especially during family time, and ensure that all family members adhere to it. The results of this study can be shared with the community.
(10) The impact of digitalization on family relationships may be communicated the public as a public service announcement, utilizing effective factors such as short films, reels, and shows concurrent patterns with.

The findings of this research go beyond being merely an academic field of interest and have clear and actionable practical implications. The findings emphasize that psychological studies may benefit from address underlying family communication and emotional needs rather than simply restricting young people’s digital habits. Programs aimed at parents show that digital self-regulation can be shaped within the framework of concepts such as family communication and harmony. It demonstrates that strong family bonds are the greatest shield against the dangers of the digital world. Ultimately, this study shows that individual and family cohesion is linked to development the ability to use technology mindfully, consciously, and judiciously, and to establish and maintain face-to-face relationships.

## Data Availability

Publicly available datasets were analyzed in this study. This data can be found here: https://www.scidb.cn/en/s/iee6nu.
